# Prediction of Key Quality Parameters in Hot Air-Dried Jujubes Based on Hyperspectral Imaging

**DOI:** 10.3390/foods14111855

**Published:** 2025-05-23

**Authors:** Quancheng Liu, Chunzhan Yu, Yuxuan Ma, Hongwei Zhang, Lei Yan, Shuxiang Fan

**Affiliations:** 1 School of Technology, Beijing Forestry University, Beijing 100083, China; liuqc@bjfu.edu.cn (Q.L.); m19213026571@163.com (Y.M.); 18701203854@163.com (H.Z.); mark_yanlei@bjfu.edu.cn (L.Y.); 2 Key Laboratory of National Forestry and Grassland Administration on Forestry Equipment and Automation, Beijing 100083, China

**Keywords:** dried jujubes, hyperspectral imaging, machine learning, deep learning, quality parameters

## Abstract

Traditional biochemical analysis methods are not only resource-intensive and time-consuming, but are increasingly inadequate for meeting the demands of modern production and quality testing. In recent years, hyperspectral imaging (HSI) technology has been widely applied as a non-destructive detection method for fruit and vegetable quality assessment. This study, based on HSI technology, systematically investigates the distribution patterns of jujube quality parameters under various drying temperature conditions. It focuses on analyzing six key quality indicators: L*, a*, b*, soluble solid content (SSC), hardness, and moisture content. HSI was used to acquire reflectance (R), absorbance (A), and Kubelka–Munk (K-M) spectral data of jujubes at various drying temperatures, followed by several spectral preprocessing methods, including standard normal variate (SNV), baseline correction (baseline), and Savitzky–Golay first derivative (SG1st). Subsequently, a nonlinear support vector regression (SVR) model was used to perform regression modeling for the six quality parameters. The results demonstrate that the SG1st preprocessing method significantly enhanced the predictive capability of the model. For the predictions of L*, a*, b*, SSC, hardness, and moisture content, the best inversion models achieved coefficients of determination $R_{p}^{2}$ of 0.9972, 0.9970, 0.9857, and 0.9972, respectively. To further enhance modeling accuracy, deep learning models such as bidirectional long short-term memory (BiLSTM), bidirectional gated recurrent unit (BiGRU), and convolutional neural network–bidirectional gated recurrent unit (CNN-BiGRU) were introduced and compared comprehensively under the optimal spectral preprocessing conditions. The results demonstrate that deep learning models significantly improved modeling accuracy, with the CNN-BiGRU model performing particularly well. Compared to the SVR model, the $R_{p}^{2}$ values for L*, a*, b*, SSC, hardness, and moisture increased by 0.005, 0.007, 0.008, 0.011, 0.007, and 0.006, respectively; the RPD values increased by 0.036, 0.04, 0.26, 0.462, 0.428, and 0.216. This study provides important insights into the further application of HSI technology in the quality monitoring and optimization of the jujube drying process.

## 1. Introduction

Ensuring continuous access to sufficient and high-quality food for the global population is fundamental to meeting basic nutritional security needs [1]. Jujube (*Zizyphus jujuba* Mill.) is one of China’s traditional specialty fruits. It is predominantly cultivated in China, which accounts for approximately 90% of the world’s annual production [2]. Jujube contains bioactive compounds such as polysaccharides, triterpenic acids, phenolics, and cyclic nucleotides, which give it high nutritional and medicinal value [3]. It is widely popular in the Chinese market, and its export constitutes a significant proportion of global production. However, jujubes are prone to rapid maturation post-harvest, and their shelf life at room temperature typically does not exceed 10 days, which limits their commercial applications, particularly during the off-season [4]. Dried jujubes, as a healthy and nutritionally rich food form, gained high consumer acceptance and are widely used in the production of preserves, dried fruits, soups, and various other food products [5,6]. Drying is a traditional method for extending the shelf life of jujubes, with approximately 80% of jujubes being processed through drying [7]. Drying methods include sun drying, hot air drying, freeze drying, microwave drying, and combined drying techniques. Among these, hot air drying is the most widely used method due to its cost-effectiveness and convenience [8,9].

However, food drying is a complex process that involves various physical and chemical transformations [10]. Improper drying treatments can result in a decline in jujube quality. Color is one of the important criteria for consumers to assess food quality, and color parameters such as L*, a*, and b* directly affect the visual appeal and market acceptance of jujubes [11,12]. Soluble solid content (SSC) is a crucial indicator of jujube sweetness and flavor. Higher SSC values lead to a sweeter taste, enhancing the appeal of jujubes to consumers [13]. Moisture content directly influences the shelf life, nutritional retention, and drying effectiveness of jujubes. Jujubes with excessive moisture are prone to mold or rot, while those with too low moisture may become dry and hard, resulting in nutrient loss, affecting texture and palatability [14]. Therefore, the ability to quickly and accurately obtain quality information during the drying process is essential for improving jujube processing quality.

Traditionally, the quality parameters of jujubes have been assessed through physicochemical analysis methods. Although these methods yield accurate results, they are often time-consuming, expensive, and destructive. Therefore, it becomes challenging to evaluate the quality of individual jujube samples efficiently and cost-effectively on a large scale [15]. Hyperspectral imaging (HSI) technology, an emerging non-destructive detection method, can simultaneously provide both spatial and spectral data of jujubes [16,17]. Due to its high sensitivity to various chemical compositions and physical characteristics, HSI technology offers more comprehensive quality information than traditional methods, significantly improving the efficiency and accuracy of food quality assessments [18]. For example, Tan, F. et al. [19] utilized visible and near-infrared (Vis-NIR) and near-infrared (NIR) hyperspectral data to successfully classify the storage duration of dried jujubes and predict quality parameters, including moisture content and total sugars during storage. Wei, Y. et al. [20] and colleagues combined HSI technology with feature wavelengths and texture features to successfully predict SSC quantitatively in Xinjiang He tian jujubes, achieving prediction results of $R_{p}^{2}$ = 0.9061, RMSEP = 0.7031, and RPD = 3.2630. Liu, Q. et al. [21] employed HSI to classify dried jujubes from fresh ones at various maturation stages and developed a partial least squares regression model based on optimal feature extraction algorithms, achieving strong prediction results for SSC and moisture. The best SSC model showed $R_{v}^{2}$ = 0.893, RMSEV = 0.819, and RPD = 3.059, while the moisture model showed $R_{v}^{2}$ = 0.903, RMSEV = 0.401, and RPD = 3.227.

To enhance the predictive capability of HSI technology, advanced modeling techniques must be integrated [22]. Data-driven modeling methods, particularly those based on machine learning, are gaining increasing importance in food drying applications. By thoroughly analyzing datasets, machine learning algorithms can accurately predict the quality parameters of food [23]. Previous studies demonstrated the potential of using machine learning algorithms in combination with hyperspectral reflectance to estimate the moisture content of jujube slices under various drying techniques, highlighting their utility as non-invasive quality control tools in food applications [24].

Deep learning, due to its ability to automatically learn effective features directly from raw data without the need for pre-extraction, shows significant advantages in handling complex datasets [25,26]. Convolutional neural networks (CNN) and recurrent neural networks (RNN) particularly demonstrated excellent performance in processing high-dimensional spectral data [27]. Long short-term memory (LSTM), a variant of RNN, is especially effective in handling long-term dependency data, as it has a stronger memory capacity and can overcome the issues of vanishing and exploding gradients, which are crucial for handling large-scale spectral data [28]. The gated recurrent unit (GRU), an improved version of LSTM, introduces gating mechanisms that reduce computational parameters, enhance data processing efficiency, and lower computational complexity during training [29]. Bidirectional long short-term memory (BiLSTM) extends LSTM by processing data in both forward and backward directions, capturing more comprehensive temporal dependencies. Similarly, the bidirectional gated recurrent unit (BiGRU) processes data in both directions, enhancing its ability to capture complex temporal patterns in sequential data.

The objectives of this study are as follows:

(1) To evaluate the distribution of jujube quality parameters (L*, a*, b*, SSC, hardness, and moisture) under varying drying temperature conditions;
(2) To acquire the reflectance spectral data of jujubes at different drying temperatures and perform spectral transformations to obtain absorbance spectra and Kubelka–Munk (K-M) spectra;
(3) To apply preprocessing methods, such as standard normal variate (SNV), baseline correction (baseline), and Savitzky–Golay first derivative (SG1st), in combination with support vector regression (SVR), to determine the optimal modeling results and preprocessing methods for different quality parameters;
(4) To apply the best preprocessing method to various deep learning models (BiLSTM, BiGRU, and CNN-BiGRU), train these models and compare their performance with that of SVR to identify the optimal predictive model, thereby enabling the prediction of jujube quality parameters.

**Figure 1:**
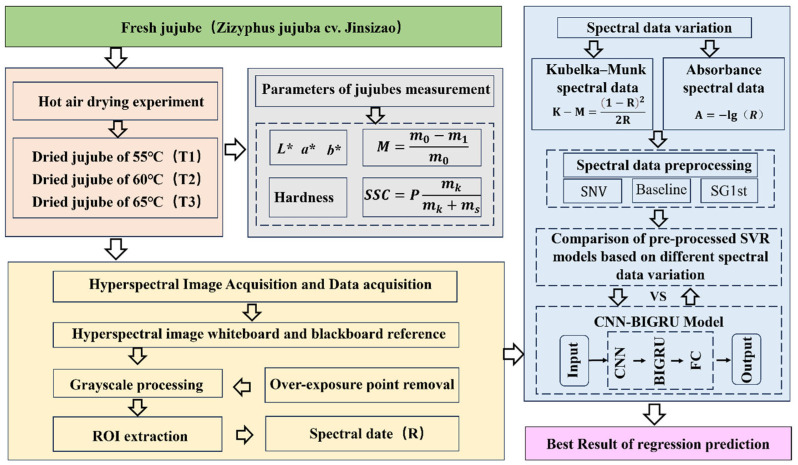
Flowchart of the hyperspectral nondestructive analysis system for hot air-dried jujubes.

The detailed workflow of the study is illustrated in Figure 1.

## 2. Materials and Methods

### 2.1. Jujube Samples and Drying Experiment

The fresh jujubes (*Zizyphus jujuba cv. Jinsizao*) were harvested in mid-October 2024 from an orchard in Le Ling City, Dezhou, Shandong Province, China (117.273° E, 37.806° N). After harvest, the fresh jujubes were transported to Beijing under low-temperature conditions at 4 °C. To ensure uniformity and minimize the influence of external factors on the drying experiment results, the maturity and size of all samples were carefully selected, and only those meeting the standards were retained. Specifically, the samples were fully mature jujubes harvested at the maturity stage, characterized by approximately 80% of the fruit surface displaying a reddish-brown color [30]. The selected samples were then randomly divided into three batches, with 90 samples per batch, totaling 270 samples. The fresh jujubes were stored in a refrigerator at 4 °C until the drying experiment began.

**Figure 2:**
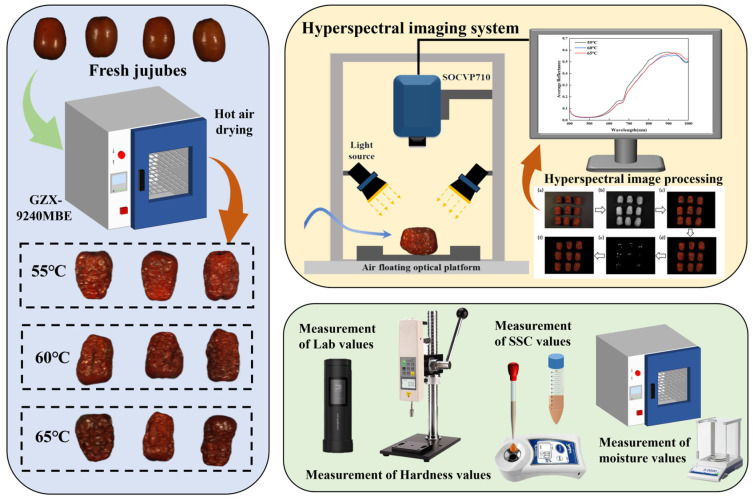
Integrated hot air drying system, hyperspectral imaging system, and the complete process scheme for quality detection.

The jujube drying process was conducted using a constant-temperature hot air drying oven (GZX-9240MBE, Shanghai Boxun Medical Biological Instrument Co., Ltd., Shanghai, China), with a temperature control range of ambient +5 °C to 200 °C, and a uniformity of ±2.5%. The fruit bed was arranged on two stainless steel mesh shelves to facilitate airflow and ensure uniform drying. A bottom-mounted fan and internal air convection system enabled bidirectional airflow, allowing samples to be dried from both faces while maintaining continuous air renewal. The drying temperature gradients were set at 55 °C, 60 °C, and 65 °C, and the air velocity inside the oven was set to 1 m/s, with a temperature precision of 0.1 °C. Prior to drying, the oven was preheated to the desired temperature and maintained for 30 min to ensure temperature stability. The jujubes were then placed on a metal mesh inside the oven, allowing the drying air to evenly remove moisture from both sides of the samples. The initial moisture content of the fresh jujubes ranged from 60% to 70%. The drying time for each sample was set to 48 h. After drying, the samples from each batch were transferred to glass drying containers and allowed to cool to room temperature (20 ± 1 °C) to minimize moisture absorption and loss during the drying process. The cooled samples were then subjected to hyperspectral image acquisition and measurements of L*, a*, b*, hardness, moisture, and SSC. The entire experimental process is shown in Figure 2.

### 2.2. Quality Parameters of Jujubes Measurement

This study measured multiple quality attributes of dried jujube samples, including L*, a*, b*, SSC, hardness, and moisture. A total of 270 jujube samples were analyzed, consisting of 90 samples for each of the three drying temperature gradients (55 °C, 60 °C, and 65 °C). Each sample was individually measured for all the above quality parameters.

**Step 1:** The color of the dried jujubes was quantified using a colorimeter (Color Meter Pro, CHNSpec Technology Co., Ltd., Hangzhou, China). For each sample, six measurements were taken along the horizontal direction at 60° intervals on the equatorial plane, and the average value was used as the color value. The L*, a*, and b* values of each sample were recorded.

**Step 2:** The hardness of de-pitted jujube samples was measured using a digital fruit hardness tester (GY-4, Eidborg Instrument Co., Ltd., Wenzhou, China). Hardness measurements were taken on the equatorial plane with six measurements at 60° intervals. A probe with a diameter of 3.5 mm and a stroke of 4–6 mm was used. The average value of the six measurements was used as the sample’s hardness value. After each measurement, the probe was cleaned and reset to zero.

**Step 3**: Moisture was determined according to the American Association of Analytical Chemists (AOAC) standard method (AOAC 1990) [31]. Jujube samples were dried in a laboratory convection oven at 105 °C for 24 h with three repeated measurements. The moisture calculation formula is as follows:

(1) $$Moisture=\frac{m_{0}-m_{1}}{m_{0}}$$

where $m_{0}$ is the weight of the specimen when tested, and $m_{1}$ is the total dry weight of the jujube samples.

**Step 4**: One gram of jujube flesh was dissolved in 10 mL of distilled water, processed by a laboratory homogenizer, and then measured for its Brix value using a digital refractometer (PR-101α, ATAGO Co., Ltd., Tokyo, Japan). The measurement result was multiplied by the dilution factor to calculate the SSC based on ISO 2173:2003 standards [32]. The SSC calculation formula is as follows:

(2) $$SSC=P\frac{m_{k}}{m_{k}+m_{s}}$$

where P is the value displayed by the digital refractometer (%), $m_{k}$ is the mass of edible parts (g), and $m_{s}$ is the mass of diluted sample (g).

### 2.3. Hyperspectral Imaging System and Date Acquisition

#### 2.3.1. Hyperspectral Imaging System

The HSI system used in this study consists of an SOC710VP hyperspectral imager (Surface Optics Co., San Diego, CA, USA), a light source, and a computer. The light source is composed of two 150 W SLS CL-150 fiber-optic halogen lamps (Technquip, Pleasanton, CA, USA), with a lamp height of 300 mm, positioned at a 45-degree angle to the sample surface. The system is housed in an external frame with dimensions of 1000 mm × 1000 mm × 1400 mm and made from 40 mm × 40 mm aluminum profiles. To minimize the interference of external ambient light on the experimental results, the frame is covered with a special light-shielding fabric, ensuring 100% light blockage. Prior to the experiment, the system was preheated for 30 min to ensure optimal spectral image acquisition within the wavelength range of 400–1000 nm. The specific parameters of the SOC710VP are shown in Table 1.

To eliminate the influence of external factors and instruments, it is necessary to correct the original hyperspectral image with white and dark reference images before extracting the spectrum. The correction formula can be represented as follows:

(3) $$R=\frac{R_{e}-R_{d}}{R_{w}-R_{d}}$$

where R is the correct hyperspectral image in units of relative reflectance (%); $R_{e}$ represents the reproduced original hyperspectral image; and $R_{d}$ is the black reference image obtained by turning off the light to block the camera lens. $R_{w}$ is the white reference image obtained from a 99% reflectance white plate.

The average reflectance (R) of the jujube within a single region of interest was calculated. Using the corresponding conversion equations, the reflectance was then transformed into absorbance (*A*) spectra and Kubelka–Munk (K–M) spectra [33]. This transformation was performed to evaluate the impact of different spectral data on the prediction of jujube quality parameters.

(4) A=−lgR

(5) $$K-M=\frac{{\left(1-R\right)}^{2}}{2R}$$

#### 2.3.2. Hyperspectral Date Acquisition and Extraction

**Figure 3:**
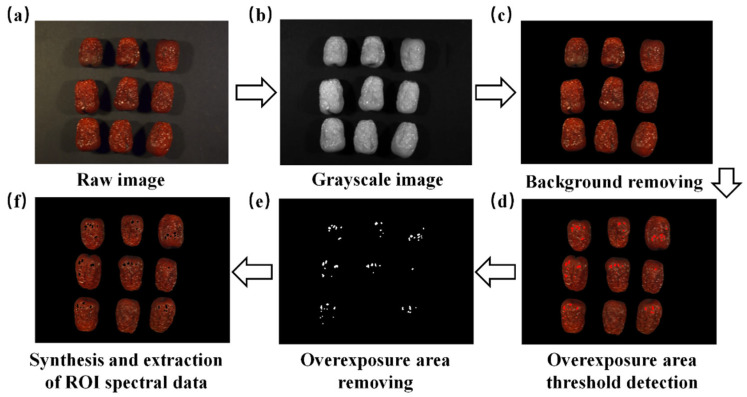
Flowchart of spectral image processing and spectral extraction. (**a**) raw image; (**b**) grayscale image; (**c**) background removing; (**d**) overexposure area threshold detection; (**e**) overexposure area removing; (**f**) synthesis and extraction of roi spectral data.

After the calibration of hyperspectral images, the spectral data of individual jujube samples were extracted. For the acquired raw images, the images were first converted into grayscale, followed by threshold segmentation to effectively separate the jujube from the background, removing the clutter and making the jujube sample more prominent. During the drying process, the skin of the jujube becomes wrinkled and irregular due to moisture evaporation, altering the light reflection patterns, particularly at the wrinkles where overexposed areas may form. To reduce the interference caused by overexposure on spectral data, threshold detection was used to remove the overexposed regions, ensuring that only the valid spectral data of the jujube were extracted. Subsequently, the entire jujube region was designated as the region of interest for spectral data extraction. Spectral data from each jujube were extracted from the selected region of interest, covering 228 bands. The entire process of spectral data collection and extraction is shown in Figure 3.

### 2.4. Spectral Preprocessing Methods

In HSI, factors such as environmental variations, electronic noise, and sample scattering frequently interfere with spectral data, impacting its reliability and accuracy. Spectral preprocessing is a crucial step in enhancing model performance, with the goal of establishing a robust model that exhibits optimal predictive capability. This study employed methods such as standard normal variate (SNV), baseline correction (baseline), and Savitzky–Golay first derivative (SG1st) for the optimization of R, A, and K-M spectral data.

SNV was employed to mitigate scattering effects in the spectral data. SNV mitigates the influence of factors such as surface scattering, particle size variations, and path length differences on reflectance spectra, thereby improving the stability and reliability of the spectral data [34]. Baseline constructs a linear correction model with 400 nm and 1000 nm as ideal baseline points, converting the skewed baseline into a horizontal one. The minimum value of the processed spectral data are then used as the baseline, and the remaining spectral values are subtracted from it, removing the influence of unfavorable factors and improving the sensitivity and accuracy of material detection [21]. SG1st applies a third-order polynomial and a seven-point smoothing operation to effectively reduce random errors in the raw spectra, thereby enhancing the signal-to-noise ratio [35]. The predictive accuracy of different preprocessing methods was compared to select the most suitable method for optimizing the performance of the jujube quality prediction model.

### 2.5. Module Establishment and Evaluation

#### 2.5.1. Support Vector Regression Module

Support vector regression (SVR) is a supervised machine learning algorithm based on the support vector machine (SVM) and is capable of performing both linear and nonlinear regression predictions [36]. The goal of SVR is to minimize the prediction error by optimizing a hyperplane while constraining the complexity of the model. The basic form of the SVR model in high-dimensional space is as follows:

(6) f(x)=w·ϕ(x)+b

where w represents the weight vector, ϕ(x) is a nonlinear mapping function that maps the input data x to a high-dimensional feature space, and b is the bias term.

SVR introduces a kernel function to map vectors from a lower-dimensional space to a higher-dimensional space, where a linear decision function is constructed, enabling nonlinear decision-making in the original space. The expression for the kernel function is as follows:

(7) $$\min\frac{1}{2}{\left\|\omega \right\|}^{2}+C\overset{N}{\underset{i=1}{\sum }}\xi_{i}+\xi_{i}^{*}$$

where ${\left\|\omega \right\|}^{2}$ is the regularization term, C is the penalty coefficient, and $\xi_{i}$ and $\xi_{i}^{*}$ are the slack variables used to measure the degree to which each data point deviates from the ϵ—epsilonϵ-insensitive margin. SVR uses an ϵ—epsilonϵ-insensitive loss function to measure the difference between the predicted values and the true values, which is expressed as follows:

(8) $$L_{\epsilon }\left(y_{i},f\left(x_{i}\right)\right)=\{\begin{array}{l} 0,\;\;\;\;\;\;\;\;\;\;\;\;\;\;\;\;\;\;\;\;\;\;\;\;\;\;\;\;\;\;\;\;if\left|y_{i}-f\left(x_{i}\right)\right|\leq \epsilon \\ \left|y_{i}-f\left(x_{i}\right)\right|-\epsilon ,\;\;\;\;\;\;\;\;\;if\left|y_{i}-f\left(x_{i}\right)\right|>\epsilon \end{array}$$

where $y_{i}$ is the actual value, $f\left(x_{i}\right)$ is the predicted value, and ϵ epsilonϵ is the allowed margin of error. To optimize the SVR model’s hyperparameters, a grid search algorithm combined with 10-fold cross-validation was used to determine the optimal combination of C and g.

#### 2.5.2. Convolutional Neural Network—Bidirectional Gated Recurrent Unit Module

**Figure 4:**
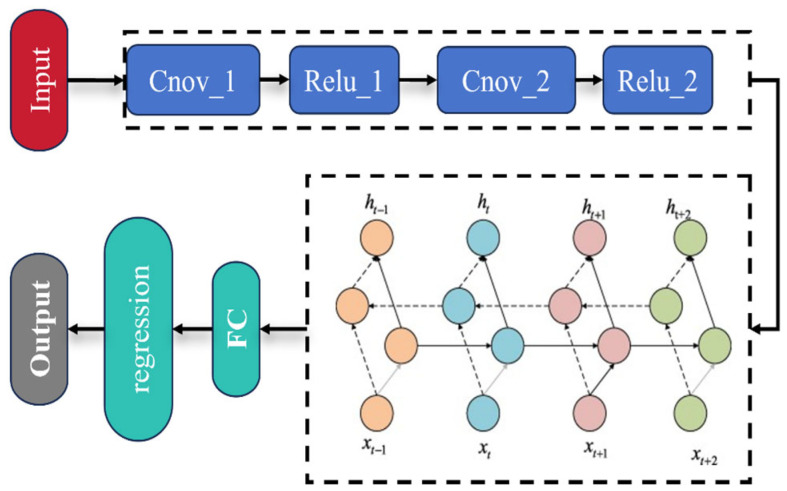
Proposed CNN-BiGRU model architecture diagram.

A convolutional neural network (CNN) is a deep feedforward neural network with convolutional computation and a deep structure. Its core lies in extracting features from input data through convolution operations. The convolutional layer’s role is to extract local features from the input data. Each convolutional layer consists of a set of small learnable convolutional kernels. In this study, two convolutional layers were set. By progressively extracting low-level features and transforming them into high-level features, the convolutional layers increase the depth of the network. To reduce the number of parameters and extract primary features, the convolved data undergo a nonlinear transformation using the rectified linear unit (ReLU) activation function. Figure 4 shows the detailed architecture of the proposed CNN-BiGRU model used.

The GRU includes update gates, reset gates, and candidate gates, which effectively solve the vanishing gradient problem by introducing gating mechanisms, thereby improving model performance. In the GRU structure, the update and reset gates are determined by the input data and interact with each other, forming specific relationships. This network’s performance is limited in practical applications because it only accesses past information, neglecting future inputs. Therefore, this study introduces BiGRU, which combines the hidden states of both forward GRU and backward GRU to generate the final output, thus fully utilizing both past and future information.

(9) $$r_{t}=\sigma \left(W_{r}x_{t}+U_{r}h_{t-1}+b_{r}\right)$$

(10) $$z_{t}=\sigma \left(W_{z}x_{t}+U_{z}h_{t-1}+b_{z}\right)$$

(11) $${\tilde{h}}_{t}=tanh\left(W_{h}x_{t}+U_{h}\left(r_{t}\cdot h_{t-1}\right)+b_{h}\right)$$

(12) $$h_{t}=\left(1-z_{t}\right)\cdot h_{t-1}+z_{t\cdot }\tilde{h_{t}}$$

where $r_{t}$ is the reset gate, $z_{t}$ is the update gate, ${\tilde{h}}_{t}$ is the candidate hidden state, σ is the sigmoid function, tanh is the hyperbolic tangent activation function, and W, U, and b represent the weight matrices and bias terms, respectively. $h_{t}$ is the output of the GRU unit.

Considering that the GRU model is a variant of the LSTM model, and that the BiGRU and BiLSTM models are derived from them, this study utilizes CNN-BiGRU, BiLSTM, and BiGRU models to predict the L*, a*, b*, SSC, hardness, and moisture of jujubes. The maximum number of training iterations was set to 500, and a grid search algorithm was employed to optimize the hyperparameters of the deep learning models. The range of hyperparameter optimization includes the number of neurons in the hidden layers (from 10 to 100), the initial learning rate (from 0.0001 to 0.01), and the L2 regularization coefficient (from 0.0001 to 0.01). By determining the optimal combination of hyperparameters, the training efficiency and prediction accuracy of the model are improved while preventing overfitting or underfitting.

#### 2.5.3. Dataset Partitioning and Model Evaluation

In this study, spectral data for 270 jujube samples were collected. To prevent data redundancy and model overfitting, the Kennard–Stone (KS) algorithm was used to divide the data into a calibration set (*n* = 190) and a prediction set (*n* = 80) in a 3:1 ratio. This method effectively selects representative sample points, ensuring the reliability and validity of model training and validation.

To comprehensively evaluate the prediction performance of different models, several commonly used evaluation metrics were employed to quantitatively analyze the model’s prediction results. Specifically, metrics such as root mean square error (RMSE), mean absolute error (MAE), mean bias error (MBE), coefficient of determination ($R^{2}$), and relative prediction deviation (RPD) were used to evaluate the model’s prediction accuracy and stability. These metrics reflect the model’s performance from multiple dimensions, ensuring that the model’s performance is fully assessed. The formulas for the evaluation metrics are as follows:

(13) $$RMSE=\sqrt{\frac{1}{N}\sum_{i=1}^{N}{\left(x_{i}-x_{i}^{\prime }\right)}^{2}}$$

(14) $$MAE=\frac{1}{N}\sum_{i=1}^{N}\left|x_{i}-x_{i}^{\prime }\right|$$

(15) $$MBE=\frac{1}{N}\sum_{i=1}^{N}\frac{\left|x_{i}-x_{i}^{\prime }\right|}{x_{i}}\times 100\%$$

(16) $$R^{2}=1-\frac{\sum_{i=1}^{N}{\left(x_{i}^{\prime }-x_{i}\right)}^{2}}{\sum_{i=1}^{N}{\left(\bar{x_{i}}-x_{i}\right)}^{2}}$$

where $x_{i}$ represents the observed value, $x_{i}^{\prime }$ represents the predicted value, $\bar{x_{i}}$ is the mean of the observed values, and N is the number of data points.

$R^{2}$ indicates the degree of fit between the model’s predicted values and the actual values. The closer $R_{p}^{2}$ is to 1, the better the model fits the data and the stronger its predictive ability. RMSE effectively evaluates the stability of the model; the smaller the RMSE, the higher the model’s accuracy. RPD is used to assess the robustness of the model, and it is the ratio of the standard deviation (SD) to the RMSE of the prediction set. Specifically, an RPD value less than 2.5 indicates poor predictive ability, an RPD between 2.5 and 3.0 indicates good predictive ability, and an RPD greater than 3.0 indicates strong predictive ability.

To further analyze the quality parameters of jujubes under different drying temperatures, Duncan’s multiple comparison test was used to perform a significance analysis of the quality parameters of dried jujubes. The quality parameters included L*, a*, b*, SSC, hardness, and moisture. Using SPSS 22.0 software, we performed statistical significance tests on multiple data groups to determine whether there were statistically significant differences in the quality of jujubes under different drying temperatures (*p* < 0.05).

## 3. Results

### 3.1. Statistics of Dired Jujube Quality Parameters

**Figure 5:**
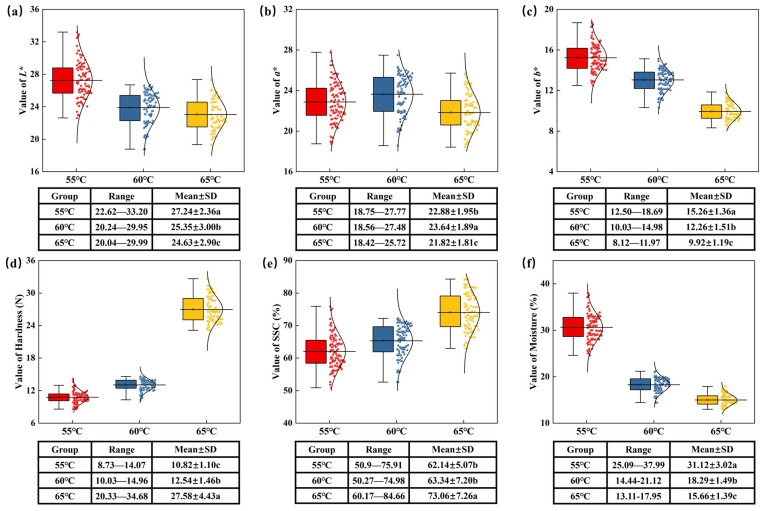
Boxplot and normal distribution of jujube quality parameters after different drying temperatures. (**a**) Distribution of L* measurement data; (**b**) distribution of a* measurement data; (**c**) distribution of b* measurement data; (**d**) distribution of SSC measurement data; (**e**) distribution of hardness measurement data; and (**f**) distribution of moisture measurement data. a–c: Different letters indicate statistically significant differences between groups, while the same letters indicate no significant difference. This notation is commonly used to summarize multiple comparison results following ANOVA.

Figure 5 presents the distribution relationships of jujube color and other quality parameters (L*, a*, b*, SSC, hardness, and moisture) at different drying temperatures (55 °C, 60 °C, and 65 °C), along with statistical data for the mean and standard deviation of each parameter. The results of the data analysis indicate that the quality attributes of jujubes exhibit significant differences in response to changes in drying temperature (*p* < 0.05).

Specifically, Figure 5a–c shows the trends of jujube color parameters L*, a*, and b* as the drying temperature changes. As the drying temperature increases, the L* value decreases significantly, indicating that the color of the jujubes darkens. The L* value was highest at 55 °C (27.24 ± 2.36), and decreased to 25.35 ± 3.00 and 24.63 ± 2.90 at 60 °C and 65 °C, respectively, indicating that the increase in temperature enhances heat-dependent reactions such as the Maillard reaction, causing the color to darken. The a* value initially increases and then decreases, reaching its highest point at 60 °C, which suggests that redness is most pronounced at this temperature—likely due to elevated temperatures promoting caramelization and pigment synthesis. These color changes may also be influenced by differences in the samples’ final moisture content. At higher drying temperatures, increased water evaporation leads to a higher concentration of solids, thereby intensifying pigment concentration and affecting color. The b* value follows a trend similar to L*, both decreasing at 65 °C, indicating a reduction in yellow components.

Figure 5d–f shows the trends of hardness, SSC, and moisture. As the temperature increases, the hardness of the jujubes increases significantly, with the highest hardness at 65 °C (22.75 ± 1.86 N) and the lowest at 55 °C (16.29 ± 1.10 N). This change is associated with moisture loss and cell component denaturation. SSC increases significantly with higher drying temperatures, reaching 27.58 ± 4.43% at 65 °C, indicating that high temperatures accelerate the concentration of SSC during moisture evaporation, retaining more nutrients. Moisture decreases significantly with increasing temperature, with 31.12 ± 3.02% at 55 °C and 15.66 ± 1.39% at 65 °C, indicating that higher drying temperatures effectively reduce the moisture of jujubes, helping to extend their shelf life and improve preservation.

### 3.2. Spectral Characteristics

**Figure 6:**
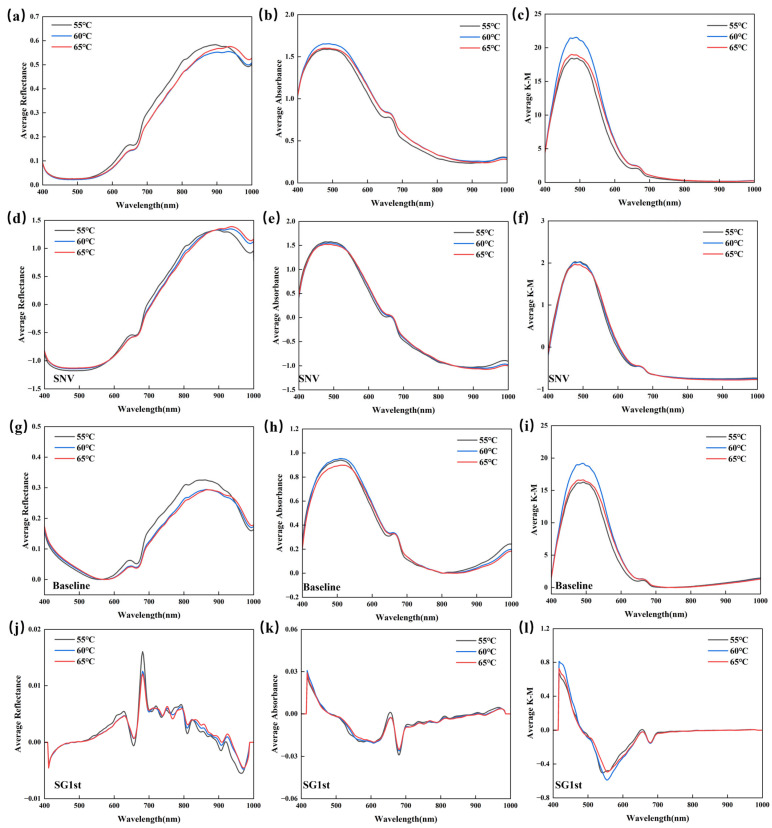
Average spectral curves of R, A, and K-M for raw and different preprocessing methods. (**a**) R raw spectral; (**b**) A raw spectral; (**c**) K-M raw spectral; (**d**) R SNV spectral; (**e**) A SNV spectral; (**f**) K-M SNV spectral; (**g**) R baseline spectral; (**h**) A baseline spectral; (**i**) K-M baseline spectral; (**j**) R SG1st spectral; (**k**) A SG1st spectral; and (**l**) K-M SG1st spectral.

Figure 6 displays the spectral characteristics of jujube samples at different drying temperatures (55 °C, 60 °C, and 65 °C), including the raw spectral data and the spectra processed with different preprocessing methods. Figure 6a–c show the raw R, A, and K-M spectra of the jujubes. As seen from Figure 6a–c, the reflectance in the 400–600 nm range for jujubes dried at 55 °C is generally higher than that for those dried at 60 °C and 65 °C, which may be related to the oxidation and degradation of carotenoids. As the drying temperature increases, the degradation of carotenoids intensifies, leading to a significant decrease in spectral reflectance. In the 600–700 nm range, there is a noticeable difference in the R, A, and K-M spectra, particularly at 650 nm and 670 nm, where small peaks and valleys appear. This phenomenon may be related to the chlorophyll content in jujubes and the C-H stretching vibration, revealing the impact of the drying process on the pigments in the jujubes. Additionally, the broad peak near 870 nm may be related to the third overtone absorption of C-H, while the absorption valleys near 920 nm and 970 nm are primarily caused by the stretching vibration of the O-H bond’s triplet frequency absorption peak in water molecules, which directly reflects the changes in the moisture of the jujubes.

Figure 6d,g,j show the R, A, and K-M spectra after preprocessing with SNV; Figure 6e,h,k shows the spectra after baseline; and Figure 6f,i,l shows the spectra after preprocessing with SG1st. It can be observed that in the high-frequency vibration 600–800 nm region of the jujube spectra, SG1st preprocessing makes the spectral details clearer, especially at the edges of the absorption peaks and valleys, helping to better identify subtle changes in the spectral features.

### 3.3. SVR Modeling Results of Different Preprocessing for R, A, and K-M Spectra

#### 3.3.1. SVR Modeling Results of Different Preprocessing for R Spectra

**Figure 7:**
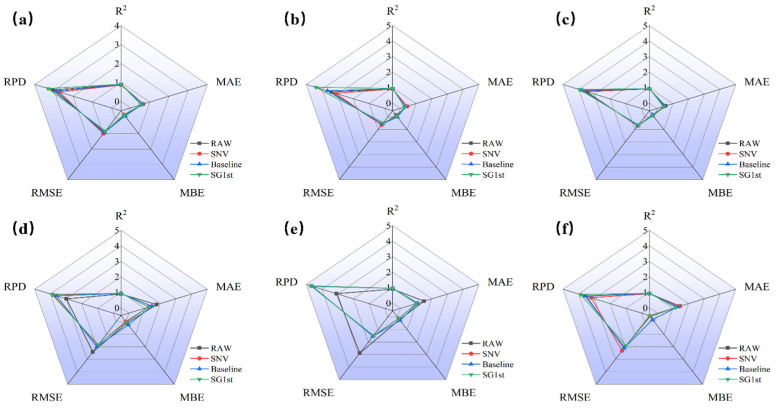
Radar distribution plot of the prediction results based on SVR for different preprocessing of R spectra. (**a**) Prediction results of L*; (**b**) prediction results of a*; (**c**) prediction results of b*; (**d**) prediction results of SSC; (**e**) prediction results of hardness; and (**f**) prediction results of moisture.

Figure 7 presents a radar chart comparing the prediction results of jujube quality parameters (L*, a*, b*, SSC, hardness, and moisture) based on R spectra with different preprocessing methods (SNV, baseline, and SG1st). Figure 7a–f shows the prediction model results for each quality parameter of jujubes. From Figure 7a–c, it can be observed that for the three parameters L*, a*, and b*, the overall trends displayed in the radar charts are consistent, indicating that the different preprocessing methods contribute similarly to improving model performance. When modeling with the raw reflectance spectral data, the $R_{p}^{2}$ values for L*, a*, and b* were 0.891, 0.913, and 0.918, respectively, and the RPD values were 3.051, 3.409, and 3.518, demonstrating good prediction performance and indicating that the raw spectral data can provide accurate predictions.

When comparing the modeling effects of different preprocessing methods, SG1st preprocessing performed the best for L*, a*, and b*. SG1st significantly increased the $R_{p}^{2}$ and RPD values, while reducing RMSE, MAE, and MBE, indicating that this preprocessing method can effectively enhance the predictive accuracy of reflectance spectral data. This result may be attributed to the advantage of the first derivative process in reducing noise and enhancing data details [37]. Tamburini, E. et al. [38] also reported that the first derivative can improve model accuracy. In contrast, SNV and baseline preprocessing resulted in a decrease in $R_{p}^{2}$ by 0.028 and 0.014, and a decrease in RPD by 0.334 and 0.177 for L*, suggesting that these preprocessing methods did not significantly improve the prediction performance of the L*, a*, and b* models.

Figure 7b–e shows the prediction results for SSC, hardness, and moisture. It can be seen that all three preprocessing methods (SNV, baseline, and SG1st) significantly improved the $R_{p}^{2}$ and RPD values for SSC and hardness, while reducing RMSE, MAE, and MBE, indicating that these methods can effectively enhance the prediction accuracy of reflectance spectral data. Particularly, SG1st preprocessing achieved the highest prediction accuracy for these parameters, with the results in Figure 7e further confirming this. In summary, the modeling results for L*, a*, b*, SSC, hardness, and moisture were optimal when using reflectance spectra combined with SG1st preprocessing. SG1st preprocessing significantly improved the model’s predictive ability, with $R_{p}^{2}$ values reaching 0.907, 0.94, 0.935, 0.934, 0.955, and 0.933, and RPD values of 3.308, 4.353, 3.954, 3.904, 4.708, and 3.883, respectively, significantly enhancing the prediction accuracy of the model.

#### 3.3.2. SVR Modeling Results of Different Preprocessing for A Spectra

**Figure 8:**
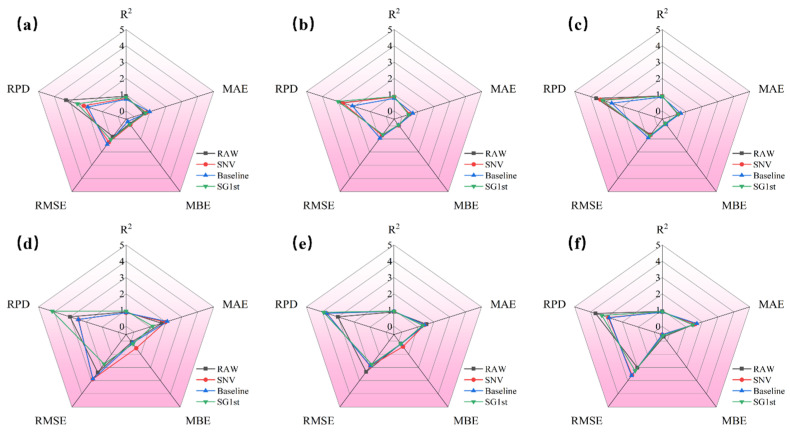
Radar distribution plot of the prediction results based on SVR for different preprocessing of A spectra. (**a**) Prediction results of L*; (**b**) prediction results of a*; (**c**) prediction results of b*; (**d**) prediction results of SSC; (**e**) prediction results of hardness; and (**f**) prediction results of moisture.

The radar chart in Figure 8 compares the prediction performance of jujube quality parameters (L*, a*, b*, SSC, hardness, and moisture) based on A spectra with different preprocessing methods (SNV, baseline, and SG1st). From Figure 8a,c–f, it can be observed that the applicability of different preprocessing methods varies for the parameters L*, b*, and moisture. For the predictions of L*, b*, and moisture, the raw spectrum without preprocessing performed the best, with $R_{p}^{2}$ values of 0.905, 0.924, and 0.925, and RPD values of 3.262, 3.641, and 3.683, respectively, while also achieving the smallest RMSE and MAE. This indicates that the raw spectral data are already sufficiently clear, and excessive preprocessing may weaken effective features, leading to a decline in model performance. This result suggests that retaining the raw data may be the optimal choice when spectral features are pronounced.

When analyzing the prediction results for b*, SSC, and hardness, the radar chart shows that SG1st preprocessing significantly improved the model’s fit, yielding the best prediction results. SG1st preprocessing performed the best for these parameters, with optimal $R_{p}^{2}$ values of 0.889, 0.941, and 0.936, and optimal RPD values of 3.024, 4.141, and 3.917, respectively, indicating that this preprocessing method can effectively enhance the prediction accuracy for these parameters.

#### 3.3.3. SVR Modeling Results of Different Preprocessing for K-M Spectra

**Figure 9:**
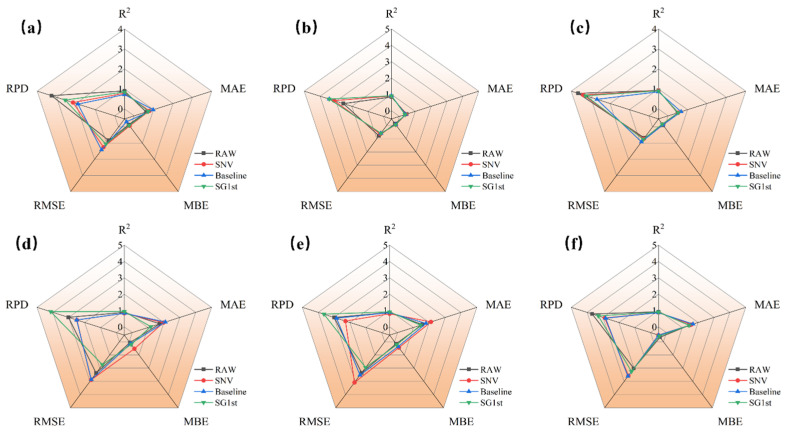
Radar distribution plot of the prediction results based on SVR for different preprocessing of K-M spectra. (**a**) Prediction results of L*; (**b**) prediction results of a*; (**c**) prediction results of b*; (**d**) prediction results of SSC; (**e**) prediction results of hardness; and (**f**) prediction results of moisture.

The radar chart in Figure 9 compares the prediction results for jujube quality parameters (L*, a*, b*, SSC, hardness, and moisture) based on K-M spectra with different preprocessing methods (SNV, baseline, and SG1st).

Figure 9a–f shows the prediction model results for each quality parameter of jujubes. From Figure 9a,c,f, it can be observed that the applicability of different preprocessing methods still varies for the L* and b* parameters. For the predictions of L* and b*, the raw spectrum without preprocessing performed the best, with $R_{p}^{2}$ values of 0.836 and 0.924, and RPD values of 2.487, 3.641, and 3.658, respectively, indicating that the raw spectral data performed optimally for these parameters.

When analyzing the prediction results for b*, SSC, hardness, and moisture, the radar chart shows that SG1st preprocessing significantly improved the model’s fit, yielding the best prediction results. SG1st preprocessing performed the best for these parameters, with optimal $R_{p}^{2}$ values of 0.914, 0.928, 0.921, and 0.935, and optimal RPD values of 3.430, 3.763, 3.642, and 3.912, respectively, indicating that this preprocessing method can significantly enhance the prediction accuracy for these parameters.

#### 3.3.4. Best SVR Modeling Results of Different Preprocessing

Based on the above analysis, the optimal spectral preprocessing method should be selected according to the specific quality parameter, rather than by applying a uniform preprocessing method. Different preprocessing methods each have their strengths and weaknesses, and they should only be used when they enhance the model’s performance. We evaluated the performance of different preprocessing methods (SNV, baseline, SG1st) in predicting quality parameters such as L*, a*, b*, SSC, hardness, and moisture of dried jujubes, and found significant differences in prediction results across the parameters [39].

**Figure 10:**
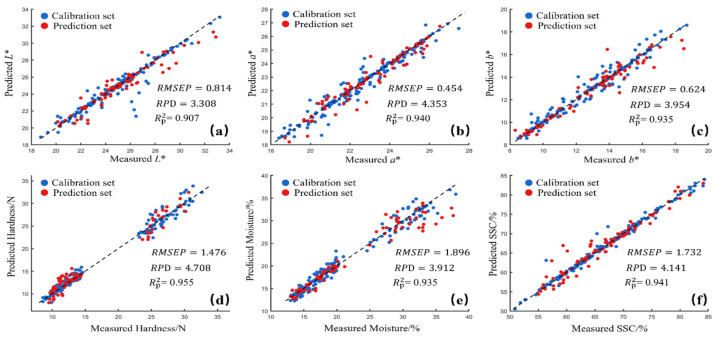
Optimal SVR-based prediction models for different quality attributes of jujube. (**a**) R + SG1st; (**b**) R + SG1st; (**c**) R + SG1st; (**d**) K-M + SG1st; (**e**) R + SG1st; and (**f**) A + SG1st.

Specifically, the SG1st preprocessing method performed excellently for most of the parameters, significantly improving the $R_{p}^{2}$ and RPD values while reducing RMSE and MAE, indicating that SG1st effectively removes noise and enhances spectral features. We compiled the optimal prediction models for each quality parameter and their corresponding preprocessing methods. The optimal prediction models for L*, a*, b*, and moisture all used the R + SG1st preprocessing method, yielding the best results: L* ($R_{p}^{2}$ = 0.907, RMSEP = 0.814, and RPD = 3.308), a* ($R_{p}^{2}$ = 0.940, RMSEP = 0.454, and RPD = 4.353), b* ($R_{p}^{2}$ = 0.935, RMSEP = 0.624, and RPD = 3.954), and moisture ($R_{p}^{2}$ = 0.935, RMSEP = 1.896, and RPD = 3.912). The regression fit curves are shown in Figure 10a, Figure 10b, Figure 10c, and Figure 10e, respectively.

For hardness, the optimal prediction model used the K-M + SG1st preprocessing method, with the following results: $R_{p}^{2}$ = 0.955, RMSEP = 1.476, and RPD = 4.708. The regression fit curve is shown in Figure 10d. For SSC, the optimal prediction model used the A + SG1st preprocessing method, yielding the following results: $R_{p}^{2}$ = 0.941, RMSEP = 1.732, and RPD = 4.141. The regression fit curve is shown in Figure 10f.

### 3.4. Result of Optimal Preprocessing on Quality Parameter Modeling in Deep Learning

**Figure 11:**
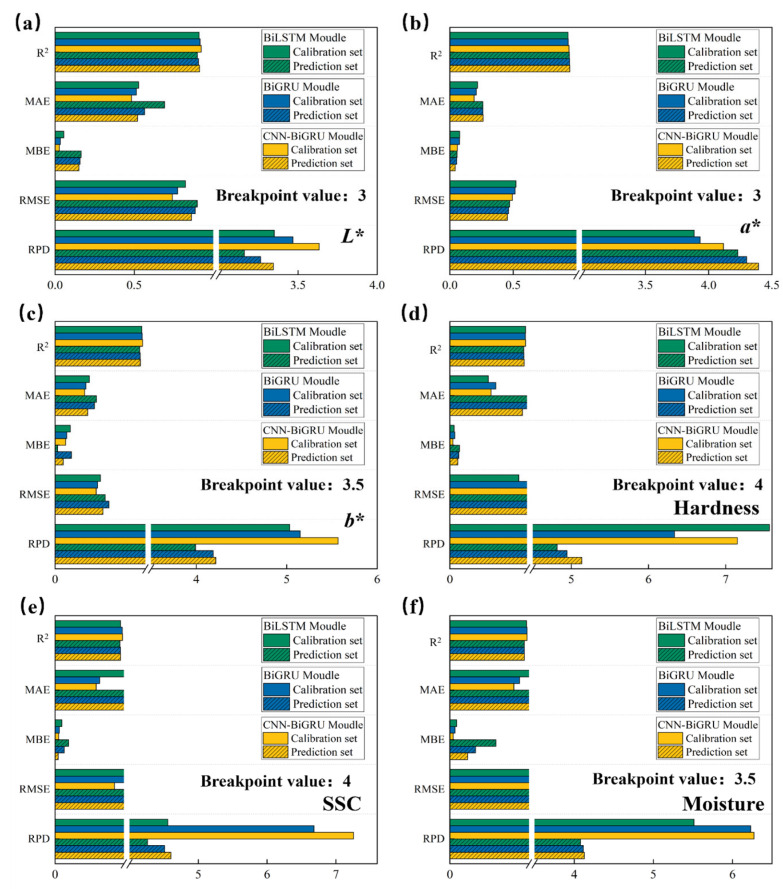
Prediction models based on BiLSTM, BiGRU, and CNN-BiGRU for different quality attributes of jujube. (**a**) R + SG1st; (**b**) R + SG1st; (**c**) R + SG1st; (**d**) K-M + SG1st; (**e**) A + SG1st; and (**f**) R + SG1st.

Figure 11 illustrates the impact of the optimal preprocessing method on the modeling results when predicting six quality parameters (L*, a*, b*, SSC, hardness, and moisture) using different models (BiLSTM, BiGRU, and CNN-BiGRU) under the deep learning model framework. The bar charts in the figure display the performance metrics of each model in the calibration and prediction sets, including $R^{2}$, MAE, MBE, RMSE, RPD, and other evaluation metrics.

From the specific evaluation results of the six different quality parameters, it is evident that the BiGRU model generally outperforms the BiLSTM model, while the CNN-BiGRU model, which combines the advantages of both CNN and BiGRU, clearly outperforms both BiGRU and BiLSTM in terms of evaluation metrics. It is noteworthy that the BiLSTM model performs worse than the SVR machine learning model in predicting L*. This may be due to the complex structure and multiple parameters of the BiLSTM model, which tends to overfit on small datasets, particularly in L* prediction. On the other hand, SVR effectively controls model complexity, improving prediction performance.

The CNN-BiGRU model shows the highest $R_{p}^{2}$ values, along with lower RMSEP and RPD values, indicating that this model can effectively capture the spectral features of these quality parameters, achieving high-precision predictions. Compared to the optimal modeling results based on SVR, the CNN-BiGRU model improved the $R_{p}^{2}$ and RPD for the L* parameter by 0.005 and 0.036, respectively; for the a* parameter by 0.007 and 0.04; for the b* parameter by 0.008 and 0.261; for the SSC parameter by 0.011 and 0.462; for the hardness parameter by 0.007 and 0.428; and for the moisture parameter by 0.006 and 0.216. In summary, deep learning models, especially the CNN-BiGRU model, demonstrated outstanding performance in handling complex spectral data and significantly enhanced the prediction accuracy for various quality parameters [40].

## 4. Discussion

HSI technology has become a research hotspot in fruit quality detection and gradually evolved into a key non-destructive tool for assessing fruit quality [41]. This study predicts six major quality parameters (L*, a*, b*, SSC, hardness, and moisture) of dried jujubes and investigates the impact of different drying temperatures (55 °C, 60 °C, and 65 °C) on jujube quality. The results indicate that drying temperature significantly affects the quality parameters of jujubes, particularly their color [42]. An increase in temperature likely triggers the Maillard reaction and other heat-dependent chemical reactions, which not only alter the color of jujubes, but may also change their final moisture content, SSC, and hardness [27]. Compared to traditional chemical analysis methods, HSI offers the advantages of being non-destructive, rapid, and efficient, making it an ideal tool for non-destructive quality detection of jujubes.

Although the results of this study are promising, several limitations remain. First, the sample size is relatively small, consisting of only 270 jujube samples. While the division into training and testing sets helped to mitigate overfitting, the limited sample size may still constrain the model’s generalization capability. Therefore, future research should employ larger and more diverse datasets to further validate the model’s stability and robustness. Moreover, future studies should expand the range of drying parameters, including varying air flow rates, temperature gradients, and other relevant factors, to achieve a more comprehensive optimization of the drying process. Integrating real-time online monitoring technologies and exploring intelligent control strategies for dynamic adjustment of drying conditions could significantly enhance the consistency and efficiency of jujube drying.

Regarding spectral data preprocessing, this study used R, A, and K-M spectra and compared three preprocessing methods: SNV, baseline, and SG1st. The results show that SG1st preprocessing performed exceptionally well in removing noise and enhancing data details. SG1st, through first-derivative transformation, preserved the trend information in the spectra, effectively reduced noise interference, and improved the model’s fit and predictive accuracy [43]. In contrast, SNV and baseline preprocessing methods did not significantly enhance the prediction performance for some quality parameters, further confirming that spectral preprocessing methods should be chosen based on the specific characteristics of the quality parameters, rather than applying a uniform strategy [44].

In deep learning model applications, the CNN-BiGRU model, which combines the advantages of CNN and BiGRU, demonstrated the best prediction performance. This model achieved the highest R^2^ values and the lowest RMSE and MAE errors, indicating that CNN-BiGRU excels in multi-dimensional feature learning and high-dimensional data feature extraction [45]. The results of this study show that deep learning models, especially in the context of multi-dimensional spectral data processing and high-dimensional feature extraction, hold immense potential for food quality detection [46]. With the appropriate spectral preprocessing methods, deep learning models significantly improve the prediction accuracy of dried jujube quality parameters, providing reliable technical support for quality control. Future research should focus on integrating advanced HSI technology and AI techniques with a deep understanding of drying systems to develop intelligent and adaptive drying processes. By incorporating real-time sensor data and process modeling, such approaches can enable dynamic control of drying parameters, enhancing efficiency, product quality, and sustainability. Building a more comprehensive food quality detection system remains a promising direction for future research.

## 5. Conclusions

This study evaluates the application potential of HSI technology for detecting six key quality parameters (L*, a*, b*, SSC, hardness, and moisture) of dried jujubes at different drying temperatures (55 °C, 60 °C, and 65 °C), comparing machine learning and deep learning models. The SG1st preprocessing method combined with the CNN-BiGRU model achieved the highest prediction accuracy, demonstrating excellent noise reduction and feature enhancement. Overall, the results highlight the promise of non-destructive, HSI technology for intelligent quality monitoring and precision agriculture.

## Figures and Tables

**Table 1:** The parameters of the SOC710VP hyperspectral imaging systems.

Parameters	Range	Parameters	Range
Wavelength range	400–1000 nm	Variables	228
Spectral resolution	4.69 nm	Dynamic range	12 bit
Speed	23.2 s/cube	Exposure time	10 -> 10^3^ ms
Pixels per line	520	Lines per cube	696

## Data Availability

The original contributions presented in this study are included in the article. Further inquiries can be directed to the corresponding authors.
